# The Anthelmintic Drug Niclosamide Induces Apoptosis, Impairs Metastasis and Reduces Immunosuppressive Cells in Breast Cancer Model

**DOI:** 10.1371/journal.pone.0085887

**Published:** 2014-01-08

**Authors:** Tinghong Ye, Ying Xiong, Yupeng Yan, Yong Xia, Xuejiao Song, Li Liu, Deliang Li, Ningyu Wang, Lidan Zhang, Yongxia Zhu, Jun Zeng, Yuquan Wei, Luoting Yu

**Affiliations:** 1 State Key Laboratory of Biotherapy and Cancer Center, West China Hospital, West China Medical School, Sichuan University, Chengdu, China; 2 College of Life Science, Sichuan University, Chengdu, China; 3 College of Chemical Engineering, Sichuan University, Chengdu, China; Rutgers - New Jersey Medical School, United States of America

## Abstract

Breast carcinoma is the most common female cancer with considerable metastatic potential. Discovery of new therapeutic approaches for treatment of metastatic breast cancer is still needed. Here, we reported our finding with niclosamide, an FDA approved anthelmintic drug. The potency of niclosamide on breast cancer was assessed *in vitro* and *in vivo*. In this investigation, we found that niclosamide showed a dramatic growth inhibition against breast cancer cell lines and induced apoptosis of 4T1 cells in a dose-dependent manner. Further, Western blot analysis demonstrated the occurrence of its apoptosis was associated with activation of Cleaved caspases-3, down-regulation of Bcl-2, Mcl-1 and Survivin. Moreover, niclosamide blocked breast cancer cells migration and invasion, and the reduction of phosphorylated STAT3^Tyr705^, phosphorylated FAK^Tyr925^ and phosphorylated Src^Tyr416^ were also observed. Furthermore, in our animal experiments, intraperitoneal administration of 20 mg/kg/d niclosamide suppressed 4T1 tumor growth without detectable toxicity. Histological and immunohistochemical analyses revealed a decrease in Ki67-positive cells, VEGF-positive cells and microvessel density (MVD) and an increase in Cleaved caspase-3-positive cells upon niclosamide. Notably, niclosamide reduced the number of myeloid-derived suppressor cells (MDSCs) in tumor tissues and blocked formation of pulmonary metastases. Taken together, these results demonstrated that niclosamide may be a promising candidate for breast cancer.

## Introduction

According to statistics, the three most commonly diagnosed types of cancer among women will be breast, lung and bronchus, and colorectum in the United States in 2013 [1]. About 64,640 cases of breast carcinoma in situ are expected to be newly diagnosed, and breast cancer is expected to account for 29% of all new carcinoma cases among women. Moreover, incidence rates are decreasing for all 4 major cancer sites (lung, colorectum, breast, and prostate) except female breast cancer, for which rates remained relatively stable from 2005 to 2009. Therefore, breast cancer ranks the second leading cause of cancer death among women worldwide, with nearly 1.4 million new cases annually [1]–[3]. Although significant progress has been made in breast cancer detection and treatment, survival tends to be poorer in economically developing countries [4]. It is critical to develop novel therapeutic approaches for the treatment of breast cancer.

It is widely known that there are some specific genetic alterations that are found in a relatively high percentage of breast cancer, such as tumor suppressor genes, cytokines, and their receptors, including Ras, c-myc, Src, Notch, Wnt/β-catenin and epidermal growth factor receptor (EGFR) [5]–[9]. Notably, these abnormalities involve the signal transducers and activators of transcription 3 (Stat3) signaling pathway [10]. In fact, the association between breast cancer and aberrant Stat3 expression was established more than 10 years ago [11], [12]. Stat3 is a point of convergence for multiple oncogenic signaling pathways. Meanwhile, Stat3 as a class of transcription factors regulates cellular and biological processes [13]. For example, activated Stat3 has been showed to promote cell proliferation and prevent apoptosis in human cancer cells by effecting misregulation of key proteins, including cell survival proteins [e.g., Mcl-1, Bcl-2 and Bcl-x_L_], inducers of angiogenesis such as hypoxia-inducible factor 1 (HIF1) and vascular endothelial growth factor (VEGF), and cell cycle regulators (e.g., cyclin D1/D2 and c-myc) [14]–[17]. In addition, studies also indicated that activated Stat3 can promote tumor cell migration and invasion and suppressed tumor-immune surveillance [18]. Furthermore, Stat3 is widely expressed in breast cancer and orally bioavailable small-molecule inhibitor of Stat3 can regress human breast cancer xenografts [12], suggesting that Stat3 is a potential therapeutic target for breast cancer. Despite the promise of a number of rational approaches to target Stat3 function and several inhibitors have been reported, so far no small molecule Stat3 inhibitor appears to be ready for clinical development [12], [19].

Niclosamide, an FDA approved anthelmintic drug, has been used to treat tapeworm infection for approximately 50 years [20]. However, niclosamide has recently been identified as a potent Stat3 inhibitor that can suppress Stat3 phosphorylation at Tyr705 and transcript activity [14], [19], [21]. Then accumulating evidence suggested that niclosamide also targeted other multiple signaling pathways (e.g., NF-kB, ROS, Notch, Wnt/β-catenin and mTORc1), most of which are closely involved with cancer stem cells [22]–[25]. Moreover, several research groups have reported that niclosamide had potent antiproliferative activity in a broad spectrum of cancer cells including solid tumor cells (e.g., head and neck cancer, non small cell lung cancer, prostate cancer, colon cancer and ovarian cancer) and hematologic cancer cell (e.g., acute myeloid leukemia) [19], [21], [26]–[28]. However, the function of niclosamide on breast cancer cells, tumor metastasis and its related molecular mechanism have not yet been systematically investigated [29].

In this study, we observed that niclosamide can inhibit proliferation, induce apoptosis and suppress cell migration and invasion in breast carcinoma cells. Moreover, it can also repress breast tumor growth and impair formation of pulmonary metastases *in vivo* by blocking angiogenesis, inducing apoptosis and reducing immunosuppressive cells. These data suggested that niclosamide may offer therapeutic benefits against metastatic breast cancer.

## Materials and Methods

### Cell Culture

Mouse breast cancer cell line 4T1, human breast cancer cell lines MDA-MB-231, MDA-MB-468 and MCF-7 were obtained from the American Type Culture Collection (ATCC, Rockville, MD, USA). Cells were propagated in DMEM or RPMI 1640 media containing 10% fetal bovine serum (FBS; Gibco, Auckland, N.Z.) and 1% antibiotics (penicillin and streptomycin) in 5% CO_2_ at 37°C.

### Reagents and Antibodies

Niclosamide was purchased from (Xiyashiji Chemical Co., LTD, ChengDu, SiChuan, China). Purity (98.5%) was measured by high-performance liquid chromatography (HPLC) analysis. For all *in vitro* studies, niclosamide was prepared initially as a 20 mM stock solution in dimethyl sulfoxide (DMSO) and stored at −20°C. Then the stock solution diluted in the relevant assay media, and 0.1% DMSO served as a vehicle control. For *in vivo* assays, niclosamide was prepared in 12.5% (v/v) aqueous Cremophor EL (CrEL) containing 2.5% (v/v) DMSO and dosed at 0.1 ml/10 g of body weight.

3-(4,5)-dimethylthiahiazo(-z-y1)-3,5-di-phenytetrazoliumromide (MTT), Triton X-100, DMSO, Hoechst 33342 were from Sigma Chemical Co.(St. Louis, MO). The primary antibodies against STAT3/P-STAT3^Tyr705^, focal adhesion kinase (FAK)/p-FAK^ Tyr925^, Src/p-Src^Tyr416^, VEGF, cleaved caspase-3 (CC-3), Bcl-2, Mcl-1, Survivin, β-actin were purchased from Cell Signaling Technology (Beverly, MA). Mouse monoclonal anti Ki-67 and Rabbit polyclonal anti-CD31 were purchased from Merck-Millipore. FITC-CD11b, PE-Gr1 conjugated antibodies were obtained from BD Biosciences. The Annexin V-FITC Apoptosis Detection Kit was purchased from KeyGen Biotech (Nan-jing, China).

### Cell Viability Assay

The cell viability of niclosamide treated breast cancer cells were determined using MTT assay as previously described, with some modification [30]. Briefly, the exponentially growing cells (3∼5×10^3^ cells/well) were seeded in 96-well plates. After 24 h incubation, the cells were treated with various concentrations of niclosamide. After treatment for 24, 48 and 72 h, respectively, the 20 µl of 5 mg/ml MTT was added to each well for 2∼4 h incubation at 37°C. The medium was subsequently discarded, and DMSO was added to dissolve the formazen. The absorbance of each well was measured at 570 nm using a Spectra MAX M5 microplate spectrophotometer (Molecular Devices, CA, USA), and the median inhibitory concentration (IC_50_) values were calculated. Each experiment was replicated at least 3 times.

### Colony Formation Assay

Cell colony formation assay was measured as previously described [31]. Briefly, 4T1 cells (400∼500 cells/well) were seeded in a 6-well plate. After 24 h incubation, the cells were treated with various concentrations of niclosamide and then cultured for another 10 days. Finally, the cells were washed with cold phosphate-buffered saline PBS, colonies were fixed with 4% paraformaldehyde and stained with a 0.5% crystal violet solution for 15–20 minutes, and the colonies (>50 cells) were counted under microscope. Data shown represents the average of three independent experiments.

### Morphological Analysis by Hoechst Staining

To identify the apoptosis induction effect of niclosamide, we analyzed the apoptosis cells by Hoechst 33342 staining. Briefly, 4T1 cells (1∼2×10^5^ cells/well) were plated onto 18-mm cover glass in a 6-well plate for 24 h. After niclosamide treatment with different concentrations for following 24 h, the cells were washed with cold PBS and fixed in 4% paraformaldehyde solution for 15 minutes. The cells were stained with the Hoechst 33342 solutions (5 µg/ml) according to the manufacturer’s instructions followed by PBS washing. Then nuclear morphology of apoptotic cells were examined under a fluorescence microscopy (Leica, DM4000B).

### Apoptotic Assay

To further confirm the apoptosis inducing effect of niclosamide, Annexin V-FITC apoptosis detection kit was used. Briefly, 4T1 cells (1∼2×10^5^ cells/well) were seeded in a 6-well plate for 24 h, and treated with niclosamide. After 24 h treatment, the cells were harvested and washed with cold PBS twice. The apoptosis levels were examined using the apoptosis detection kit according to manufacturer’s instructions by flow cytometry (FCM) (BD Biosciences). Then data were analyzed with FlowJo software.

### Flow Cytometry

We prepared single-cell suspensions of tumor by enzymatic dispersion as described previously [32]. Then 1×10^6^ freshly prepared cells were suspended in 100 µl PBS and stained with combination of fluorochrome-coupled antibodies to CD11b and Gr1. Cells were collected by FCM. Data were analyzed with FlowJo software.

### Western Blot Analysis

The western blot analysis was performed as described previously, with some modifications [33]. Briefly, 4T1 cells were treated with niclosamide in designed concentration for 24 hours, then cells were washed with cold PBS twice and lysed in RIPA buffer. Protein concentrations were measured using the Lowry method and equalized before loading. Equal amounts of total protein from each sample was applied to sodium dodecyl sulfate-polyacrylamide gel electrophoresis (SDS-PAGE) gels and transferred onto polyvinylidene difluoride (PVDF) membranes (Amersham Bioscience, Piscataway, NJ). After electrophoresis, the membranes were blocked for 2 h at 37°C and incubated with specific primary antibodies overnight at 4°C, followed by the secondary antibody conjugated to horseradish peroxidase. The reactive bands were detected using a commercially available enhanced chemiluminescence kit (Amersham, Piscataway, NJ).

### Boyden Chamber Migration and Invasion Assay

Boyden chamber (8 µm pore size) migration assay was performed as previously described, with some modification [34]. Briefly, 5×10^4^ 4T1 cells or MDA-MB-231 cells in 100 µl serum-free medium were added in the top chamber, then 600 µl of medium with 10% FBS was added to the bottom chamber. Different concentrations of niclosamide were added in both chambers. Cells were allowed to migrate for 20 hours. Non-migrated cells in the top chamber were removed. The migrated cells were fixed in 4% paraformaldehyde and stained with 0.5% crystal violet. Migrated cells were counted and photographed under a light microscope. Invasion assay was conducted according to previous study [34]. Briefly, the upper surface of the transwell plate was coated with 60 µl Matrigel (BD Biosciences). After Matrigel polymerization, the bottom chambers were filled with 500 µl medium containing 10% FBS. 5×10^4^ 4T1 cells or MDA-MB-231 cells in 100 µl serum-free medium were added in the upper part of each transwell and treated with different concentrations of niclosamide. After incubation for 20 hours, non-migrated cells on the top side of the filter were removed, and migrated cells were fixed with 100% methanol and stained with 0.5% crystal violet, then migrated cells were counted and photographed under a light microscope. Percentage of migrated cells inhibited by niclosamide was quantified.

### Mice and Tumor Model

All animal experiments were approved and conducted by the Institutional Animal Care and Treatment Committee of Sichuan University in China (Permit Number: 20121101). Female BALB/c mice (Six- to eight-week-old) used in this study were obtained from Beijing HFK bioscience CO. Ltd, Beijing, China. Briefly, 100 µL 4T1 tumor cell suspension containing 1.0×10^6^ cells were injected subcutaneously in the right flank of BALB/c mice. About seven days after inoculation tumor cells, the tumor-bearing mice were randomized into three groups (8 mice per group), and received intraperitoneally injection (i.p.) of niclosamide 20 mg/kg, 10 mg/kg or vehicle, respectively once daily for 21 days. Tumor volumes and body weight were assessed every three days. The tumor size was calculated according to the formula: Tumor volume (mm^3^) = 0.52×*L*×*W* ^2^ where *L* is the length and *W* is the width. Moreover, when all animals were euthanized by cervical dislocation, the lungs were harvested, total number of lung metastases was counted.

### Immunohistochemistry

Immunohistochemistry staining of tumor sections were described previously [18]. One part of paraffin tumor sections was stained with hematoxylin and eosin (H&E). The other part was stained with Ki67, cleaved casepase-3, VEGF antibodies using immunohistochemistry staining to investigate tumor cell proliferation and apoptosis, respectively. In addition, paraffin-embeded tumor sections were stained with an anti-CD31 antibody to examine blood vessel density. Images were taken with Leica microscope (Leica, DM4000B).

### Toxicity Evaluation

To test potential side effects or toxicity on mice during the treatment, all the animals were observed continuously for relevant indexes such as body weight, anorexia, diarrhea and other clinical symptoms. At the 28th day, all animals were euthanized by cervical dislocation after taking blood from eyeball. Blood was obtained for blood routine analysis by Nihon Kohden MEK-5216K Automatic Hematology Analyzer. The tissues of heart, lung, spleen, liver and kidney were stained with H&E for histopathologic examination.

### Statistical Analysis

Data represented as means±SD of three independent experiments. The statistical comparisons were made by Student’s T test and statistically significant *p* values were labeled as follows: *P<0.05; **P<0.01; ***P<0.001.

## Results

### The Anti-proliferation Effects of Niclosamide against Breast Cancer Cells

**Figure 1:**
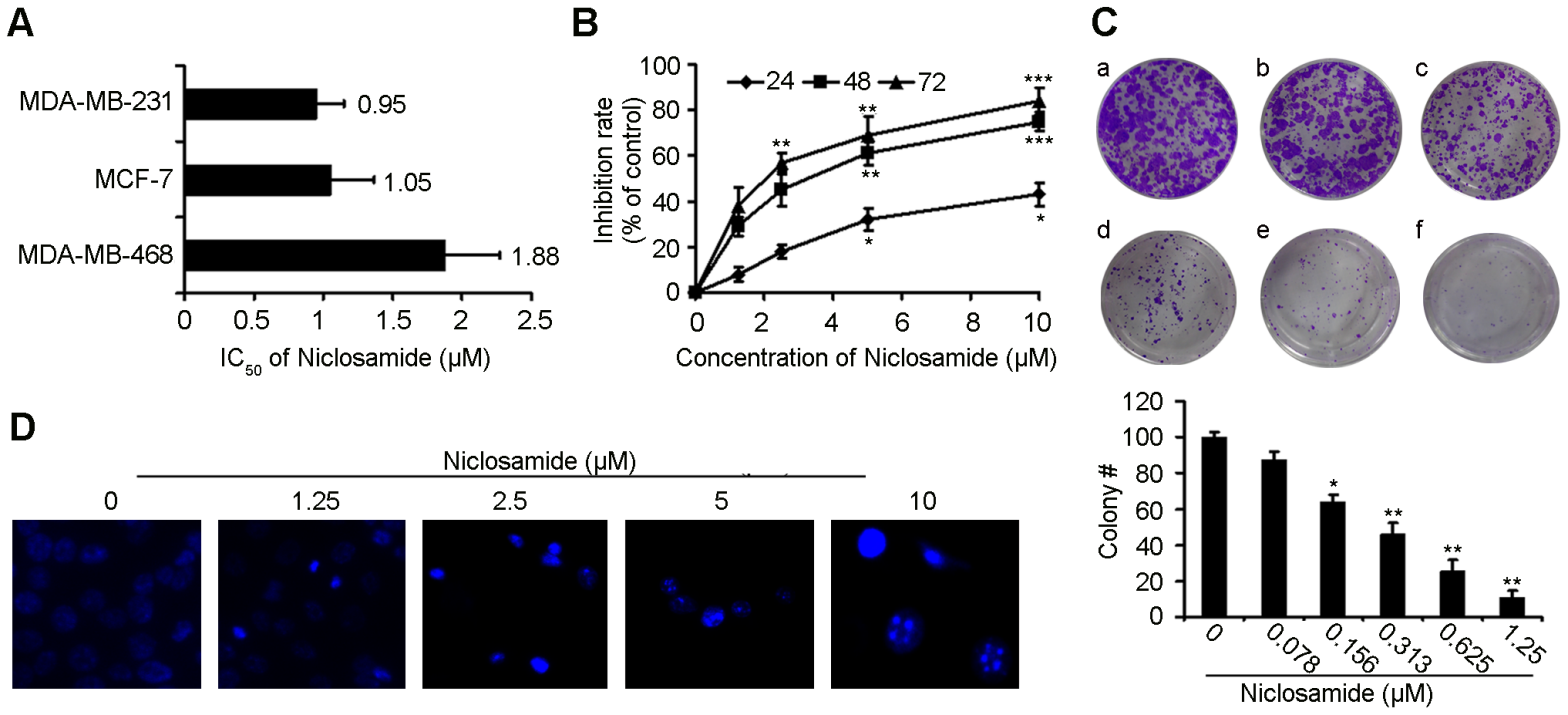
The effect of niclosamide on breast cancer cells viability. (A) Proliferation of MDA-MB-231, MCF-7, MDA-MB-468 cells treated with various concentrations (0–10 µM) of niclosamide for 72 hours, respectively. Cell viability was detected by MTT assay. The data are expressed as the means ± SD from three independent experiments. (B) MTT assays showed niclosamide inhibited 4T1 breast cancer cells proliferation concentration- and time-dependently. Values represented means ± SD from three experiments (*p<0.05; **p<0.01; ***p<0.001). (C) The effects of niclosamide (a-f:0–1.25 µM) on colony formation in 4T1 cells 12 days, the statistic results of colony formation assays presented as surviving colonies. Data are expressed as means ± SD from three experiments (*p<0.05; **p<0.01). (D) The fluorescence microscopic appearance of Hoechst 33342 staining nuclei of 4T1 cells with various concentration niclosamide for 24 h (40×). Data are the representative from three parallel experiments.

In order to investigate whether niclosamide has direct effects on breast cancer cells, we tested the proliferation inhibition caused by niclosamide treatment on different breast cancer cell lines by MTT. After exposure to niclosamide for 72 h, the IC_50_ of MDA-MB-231, MCF-7, MDA-MB-468 were 0.95 µM, 1.05 µM and 1.88 µM, respectively (Figure 1A). Exposure of 4T1 cells to niclosamide for 24 h, 48 h and 72 h, respectively, resulted in decrease of the cell proliferation (Figure 1B). Therefore, these results demonstrated that niclosamide inhibited breast cancer cells proliferation in a time- and concentration-dependent manner. Thus, we chose 4T1 and MDA-MB-231 cell lines for further experiments.

To further determine whether niclosamide could inhibit the proliferation of 4T1, we conducted colony formation assay after niclosamide treatment. As shown in Figure 1C, clonogenic assay clearly showed that clone formation of 4T1 cells was reduced in a concentration-dependent manner after exposure to niclosamide. Furthermore, the size of the colonies treated with niclosamide was significantly smaller than the control.

### Induction of Apoptosis by Niclosamide

**Figure 2:**
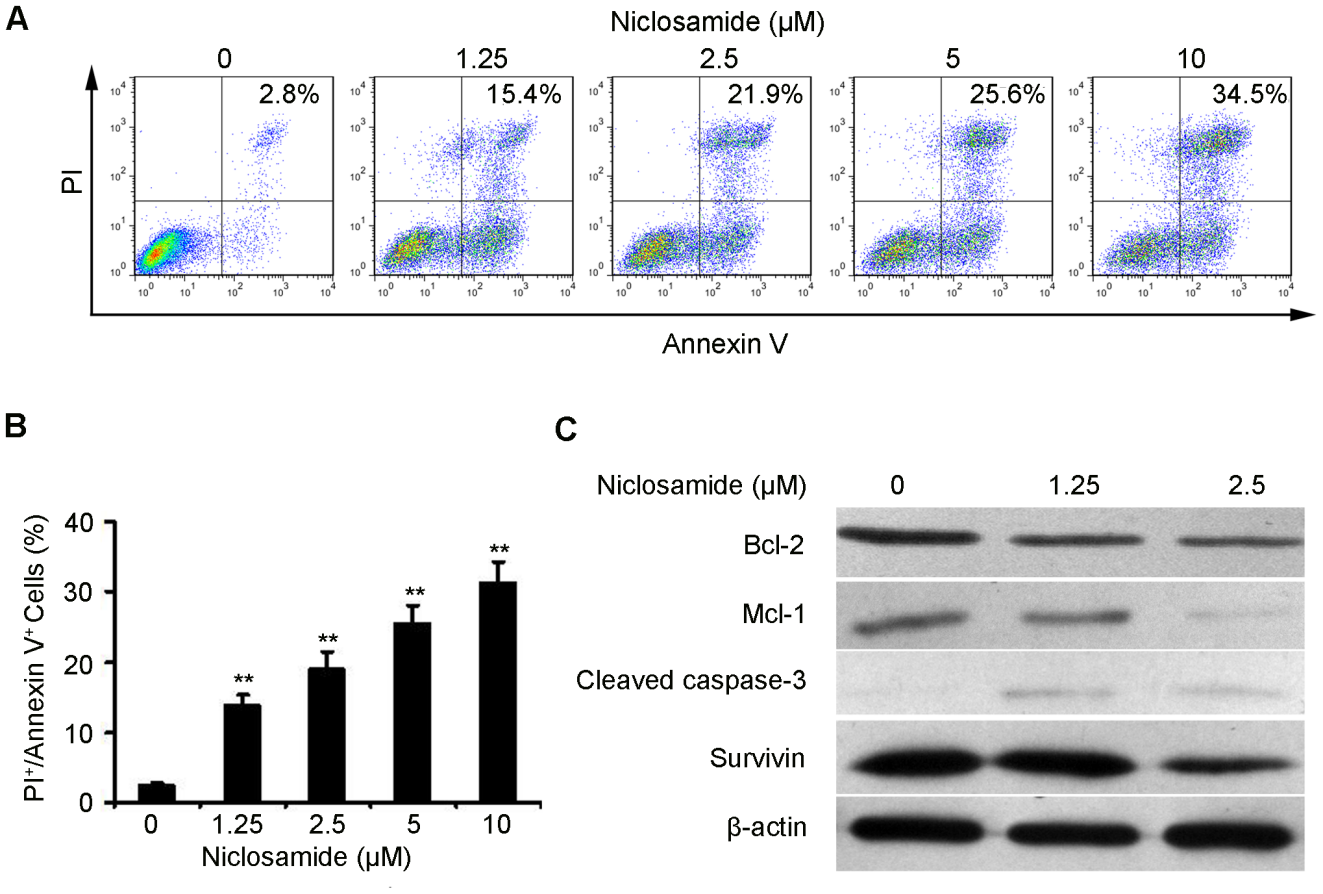
Niclosamid induces 4T1 breast cancer cells apoptosis. (A) 4T1 cells were treated with niclosamide at indicated doses for 24 hours, and the level of apoptosis was evaluated using the Annexin V/PI dual-labeling technique, as determined by FCM. Data shown are representative of three independent experiments. (B) Statistic results of apoptosis assays, 4T1 cells positive for both Annexin V and PI were considered apoptotic. Data are expressed as means ± SD from three independent experiments (*p<0.05; **p<0.01). (C) Western blot analyses of 4T1 cells treated (24 h) with different concentrations of niclosamide to evaluate protein expression of Bcl-2, Mcl-1, Cleaved caspase-3, Survivin and β-actin was employed as a standard.

As Figure 1D indicates, the 4T1 cells exhibited features of apoptosis as showed by Hoechst 33342 staining, such as bright-blue fluorescent condensed nuclei, nuclear fragmentation and reduction of cell volume. To further confirm the induction of apoptosis in 4T1 cells with niclosamide treatment, we also investigated the levels of apoptosis using the AnnexinV-FITC/PI dual-labeling technique by FCM. As shown in Figure 2A and B, after niclosamide treatment for 24 h, the apoptosis induction effect was apparently observed. When the 4T1 cells were treated with 1.25 µM niclosamide, the apoptosis rate was 13.7%, whereas the apoptosis cells increased to 19.0%, 25.5% and 31.3% when cells were treated with 2.5 µM, 5 µM and 10 µM niclosamide, respectively. Moreover, we examined Bcl-2, Mcl-1, Survivin and cleaved caspase-3 expression levels in 4T1 cells after niclosamide-treated for 24 h by western blotting analysis. The expression of Bcl-2, Mcl-1 and Survivin significantly decreased while that of cleaved caspase-3 increased in a concentration-dependent manner (Figure 2C), which was coincident with the results of Hoechst 33342 staining and FCM assays.

### Niclosamide Suppresses Breast Cancer Cell Migration and Invasion

**Figure 3:**
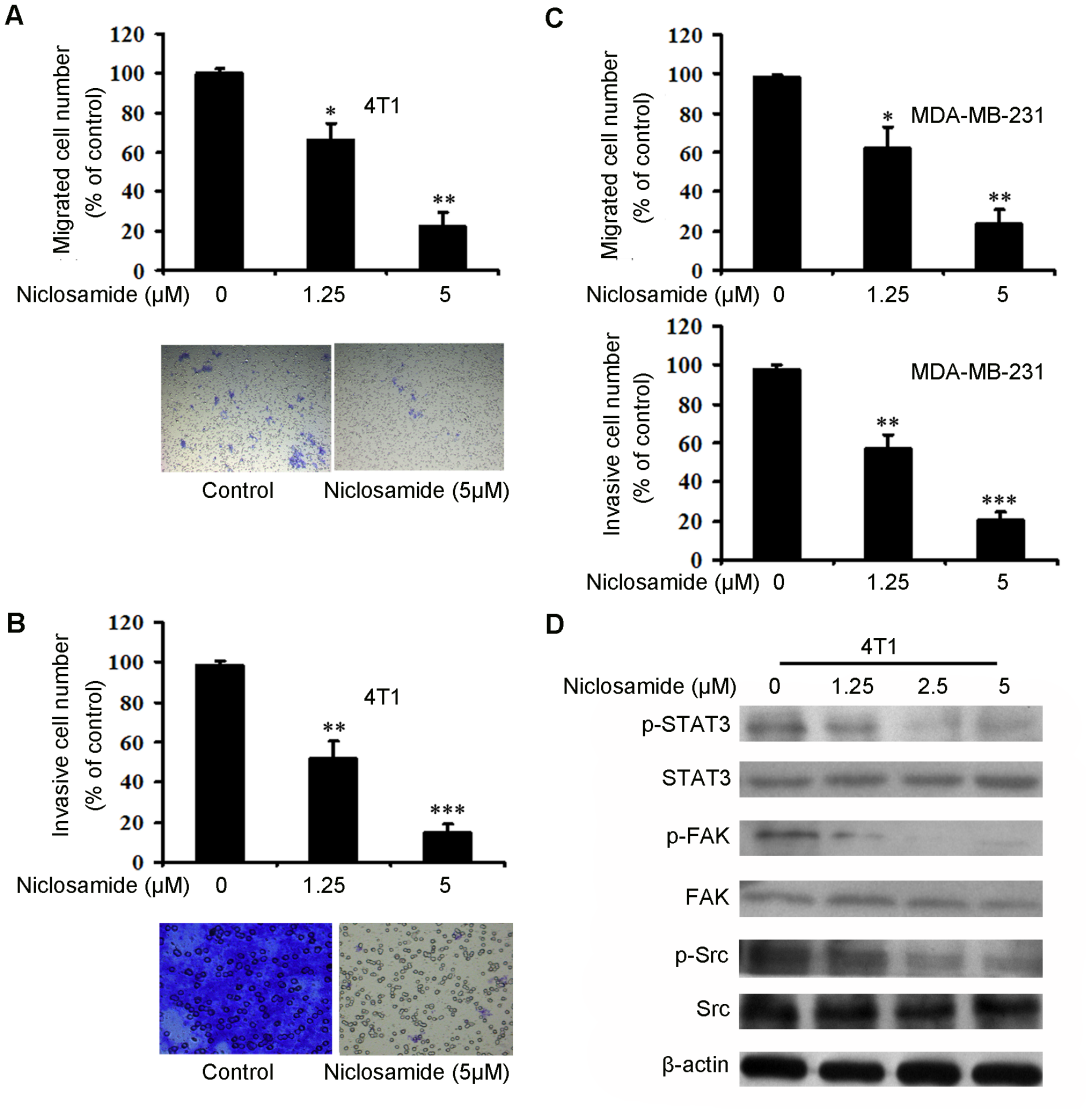
Niclosamide inhibits breast cancer cell 4T1 and MDA-MB-231 migration and invasion and inhibits FAK-involved pathway. (A) A total of 5×10^4^ 4T1 cells were seeded in the top chamber of transwell with serum-free medium, and treated with different concentration of niclosamide. After 20 hours, migrated cells were stained, photographed (10×) and quantified. (B) A total of 5×10^4^ 4T1 cells were treated with various concentration of niclosamide and allowed to invade through Matrigel and Transwell membrance, Invaded cell number was stained, photographed (20×) and counted. (C) Niclosamide inhibited MDA-MB-231 migation and invasion. The number of migrated cells and invaded cells was counted, respectively. Data represent means ±SD. (n = 3, in triplicate; *p<0.05; **p<0.01; ***p<0.001). (D) 4T1 cells were treated with different concentration of niclosamide (0–5 µM). After 24 hours, cell lysates were blotted with spectfic antibodies (anti-phospho-STAT3, anti-phospho-FAK, anti-phospho-Src, STAT3, FAK and Src), β-actin was the loading control.

Breast cancer metastasis poses a predominant threat to cancer related mortality. Moreover, one of the key steps in successful cancer metastasis is tumor cell migration and invasion [34], [35]. Therefore, in order to examine whether niclosamide could inhibit breast cancer cell migration and invasion, we performed transwell migration and invasion assays on 4T1 and MDA-MB-231 cell lines. As shown in Figure 3A, niclosamide-treated groups showed reduced migrated cell numbers on 4T1 cells, similar results was obtained in invasion assay (Figure 3B). Meanwhile, niclosamide obviously inhibited MDA-MB-231 migration and invasion were also observed (Figure 3C). Moreover, we also investigated whether Stat3, Focal Adhesion Kinase (FAK) and Src, which are considered to be related with cell migration and invasion, are involved in niclosamide-mediated migration and invasion [34]. As Figure 3D indicates, niclosamide treatment decreased the expression of phosphorylated-STAT3 ^(Tyr705)^, phosphorylated-FAK ^(Tyr925)^ and phosphorylated-Src ^(Tyr416)^ without affecting their total expression level. Taken together, these results suggested that niclosamide could suppress breast cancer cell migration and invasion in a concentration-dependent manner.

### Anti-tumor Efficacy of Niclosamide in 4T1 Mouse Mammary Tumor Model

**Figure 4:**
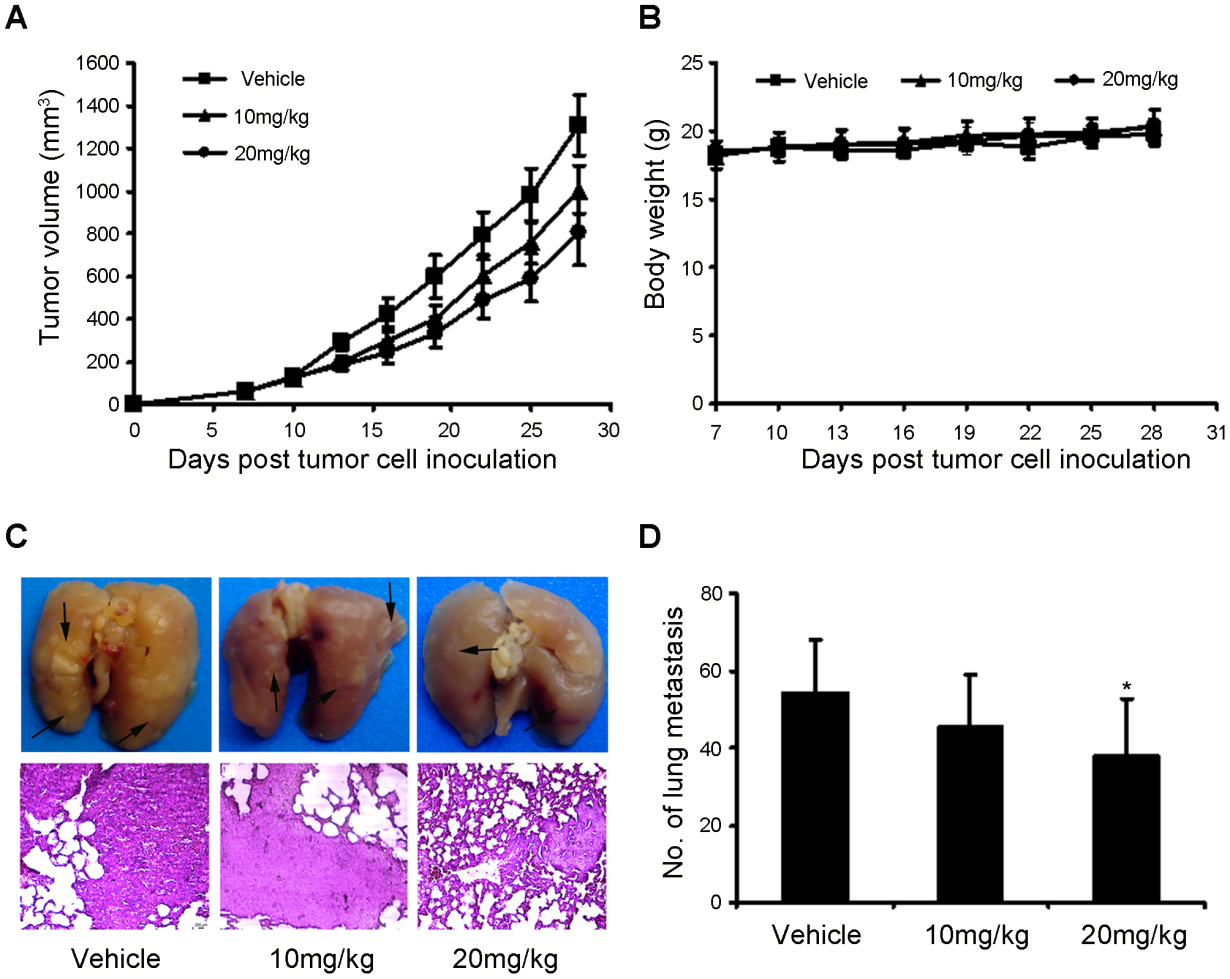
Effect of niclosamide treatment on primary tumor growth and pulmonary metastasis. (A) 4T1 tumor-bearing female BALB/c mice were treated as described with vehicle, niclosamide at 10 and 20 mg/kg, the mean tumor volumes ± SD of six mice per every group. (B) After 28 days of tumor cell inoculation, the body weight of the niclosamide treatment and vehicle groups were statisticed, and there were no significant difference among the groups. (C) Lung metastatic nodules were visualized to show the inhibitory effect of niclosamide on 4T1 tumor 21 days after treatment. Arrow indicated metastatic nodules (up), The H&E staining of lungs from each group (10×). (D) The mean lung metastasis nodules of each group, the treatment with niclosamide at 20 mg/kg resulted in significant inhibition of lung metastasis versus vehicle control. Bars showed means ± SD (n = 6; *P<0.05).

To study the antitumor activity of niclosamide *in vivo*, 4T1 tumor-bearing mice were treated with niclosamide at the dose of 10 mg/kg and 20 mg/kg. From the results (Figure 4A), it was found that the tumor growth of the niclosamide groups become slowed 7 days after treatment. After 21 days treatment, niclosamide substantially suppressed tumor growth in a dose-dependent manner compared with the control. Moreover, after treatment with niclosamide for 21 days, body weight of the mice were statisticed and no significant differences in body weight were found among the three groups (Figure 4B). Furthermore, previous studies have showed that 4T1 mouse breast cancer have a high metastatic potential and spontaneously metastasize to secondary foci from the primary sites and one of the fatal metastatic organs is lung as early as 2 weeks after inoculation [36], [37]. In the present study, we seek to evaluate whether treatment of niclosamide could reduce the occurrence of lung metastasis. The data in Figure 4C and D showed that niclosamide-treated at 20 mg/kg resulted in significant reduction in the number of lung metastases compared with other groups. In addition, histological analyses proved that the number of micrometastatic nodules per field in the niclosamide-treated at 20 mg/kg group was also significant fewer than other groups (Figure 4C). These results further indicated that high dose of niclosamide could inhibit tumor metastasis in breast cancer.

### Niclosamide Reduces the Number of Tumor-infiltrating Gr1^+^ CD11b^+^ MDSCs

**Figure 5:**
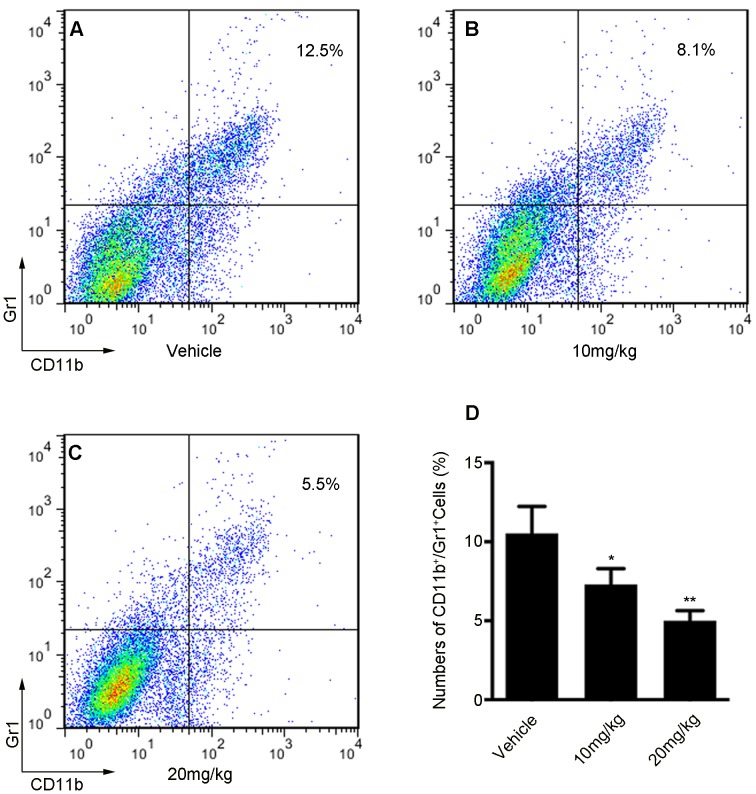
Effect of niclosamide treatment on tumor-associated Gr1^+^ CD11b^+^ MDSCs. Gr1^+^ CD11b^+^ cells were gated and analyzed by FCM for the expression of MDSCs. MDSCs isolated from the tumor tissue of 4T1 tumor-bearing mice were treated with vehicle (A); or treated with niclosamide at 10 mg/kg (B); or treated with niclosamide at 20 mg/kg (C). (D) Statistic results of each group. Treatment of niclosamide significantly reduces the number of MDSCs compared with vehicle group. Values represented means ± SD (n = 6; *p<0.05; **p<0.01).

Previous study identified Stat3 as a critical molecule in myeloid derived suppressor cells (MDSCs) accumulation in tumor-bearing mice [38]. In addition, the excess of MDSCs can promote tumor growth and immune evasion. Moreover, MDSCs have been closely related to a lung metastatic in patients with breast cancer [39].As niclosamide inhibited Stat3 signaling pathway in breast cancer cells, we further investigated whether it also reduced the number of tumor-infiltrating MDSCs (Gr1^+^ CD11b^+^). As shown in Figure 5A, B and C, the FCM data exhibited that the number of MDSCs decreased in the 10 mg/kg niclosamide-treated group compared with vehicle treated group. Moreover, we also found that 20 mg/kg niclosamide-treated further reduced the number of MDSCs compared with other groups. Importantly, statistical analysis also demonstrated that niclosamide-treated reduced the number of MDSCs in a dose-dependent manner (Figure 5D). These results implied that reducing tumor-infiltrating MDSCs could improve the treatment for breast cancer.

### Immunohistochemistry Assay

**Figure 6:**
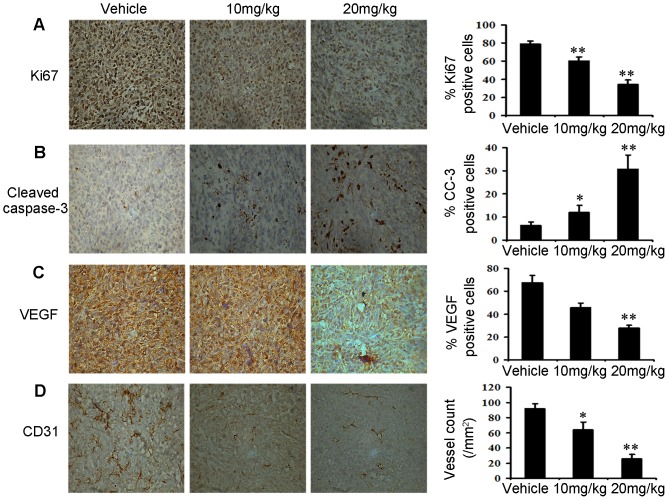
Niclosamide reduces tumor cell proliferation, induces tumor apoptosis and inhibits tumor angiogenesis in vivo. (A) Tumor cell proliferation was evaluated on paraffin-embedded 4T1 tumor sections by Ki67 immunohistochemical staining (40×), 4T1 tumor tissues removed after 21 days treatment. The treatment with niclosamide resulted in markedly reduced proliferation versus vehicle group (n = 5; **p<0.01). (B) Apoptosis was measured on paraffin-embedded 4T1 tumor sections by Cleaved caspase-3 (CC3) immunohistochemical staining (40×). The apoptosis index was calculated by dividing the number of CC3-positive cells by the total number of cells. The treatment with niclosamide significantly increased apoptosis in a dose-dependent manner compared with vehicle group (n = 5; *p<0.05; **p<0.01). (C) Immunohistochemistry was performed to measure the expression of VEGF in tumor tissues isolated from vehicle and niclosamide-treated mice (40×). The treatment with niclosamide markedly reduced VEGF-positive cells versus vehicle group (n = 5; **p<0.01). (D) Niclosamide significantly inhibited tumor vessels in 4T1 tumor. Paraffin-embedded of 4T1 tumor sections were tested by immunohistochemical analysis with anti-CD31 antibody. Representative tumor vasculature from vehicle- and niclosamide-treated mice was shown (40×). The density of microvessel was calculated in each group (n = 5; *p<0.05; **p<0.01).

To define the mechanisms through which niclosamide elicited 4T1 tumor growth inhibition *in vivo*, histological and immunohistochemical analyses were performed on tumor tissues. As Figure 6A and B showed that niclosamide treatment caused a significant reduction in the number of Ki67-positive cells, but a marked increase in the number of cleaved caspase-3-positive cells compared with the vehicle-treated group. These results indicated that antiproliferation and induction of apoptosis activity of niclosamide *in vivo* was consistent with results that niclosamide inhibited 4T1 cancer cells proliferation and induced apoptosis *in vitro*. Moreover, VEGF has been associated with tumor angiogenesis and poor prognosis [40]. We sought to measure the effects of niclosamide on angiogenesis by measuring the expression of VEGF in tumor cells by immunohistochemistry analysis. Treatment of mice with niclosamide resulted in inhibition in tumor tissues compared to the vehicle mice (Figure 6C). Furthermore, Immunohistochemical anti-CD31 staining of the tumor tissues from niclosamide-treated mice showed significantly decreased microvessel density compared with vehicle treated group (Figure 6D).

### Toxicity Evaluation

**Figure 7:**
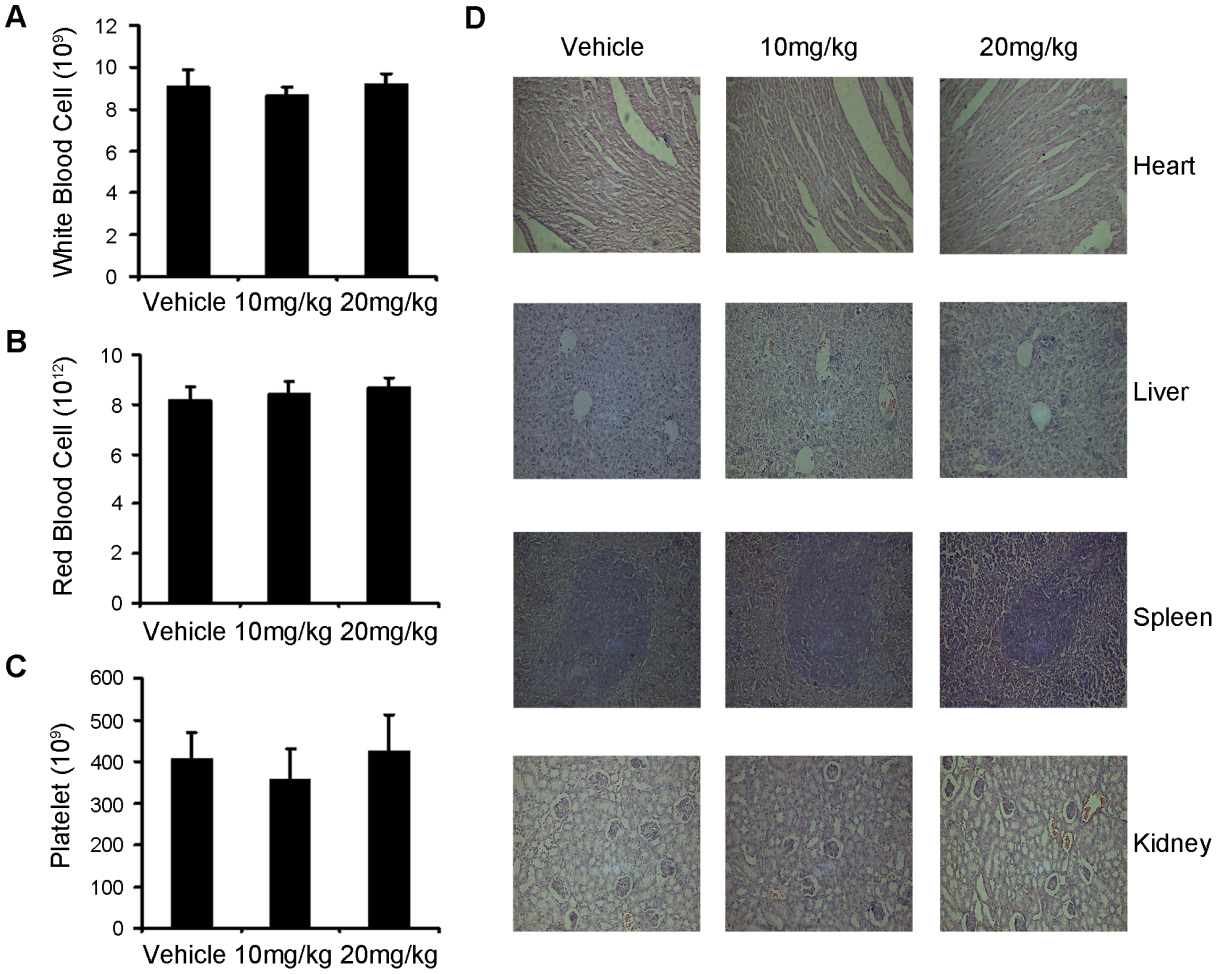
Evaluation of side effects of niclosamide in mice. There were no significant different among the vehicele-treated group and the niclosamide-treated groups in blood routine analysis of toxicity test, (A) Red blood cell. (B) White blood cell. (C) Platelet. (D) Niclosamide did not casue obvious pathologic abnormalities in normal tissues. H&E staining of paraffin-embedded sections of the heart, liver, spleen and kidney (20×).

As mentioned above, mice treated with niclosamide for 21 days showed no adverse effects in gross measures such as toxic death, skin ulceration and body weight loss. To further investigate the safety of niclosamide, we determined here whether the niclosamide could cause the blood system’s abnormality, we performed blood routine analysis assay. The data in Figure 7A, B and C showed that mice treated with niclosamide did not show significant difference compared with those of vehicle group. There were certain fluctuations among the groups, but they were in the range of normal values. Furthermore, no pathologic changes were observed in the heart, liver, spleen, lungs and kidneys by microscopic examination (Figure 7D).

## Discussion

Breast cancer is highly malignant with considerable metastatic potential, which urges to develop novel potential drug candidate to prevent tumor metastasis and treat tumor growth at present [34]. Stat3 has multiple functions and appears to play a key role in the development of breast tumor formation. Importantly, Stat3 phosphorylation (Tyr705) was observed in nuclei in approximately 35% of breast cancer tissues [41]. In addition, Stat3 also up-regulates other important downstream genes that contribute to cell proliferation, cell survival and angiogenesis in breast cancer [13], [41]. In the present study, our results showed that niclosamide, a new potent inhibitor of Stat3 signaling pathway, decreased expression of Stat3 phosphorylation at tyrsione residue 705 in 4T1 mouse mammary cancer cells (Figure 3D). Moreover, the *in vitro* studies also demonstrated that niclosamide can inhibit breast cancer cell vitality with low micromole. Furthermore, the results of Hoechst staining and FCM assays demonstrated that niclosamide induced apoptotic death in breast cancer cells in a concentration-dependent manner, which was further confirmed by the down-regulation of Bcl-2 and the up-regulation of cleaved caspase-3.

Some compounds exhibit potent antitumor activities *in vitro* but show no activity of antitumor *in vivo*. Therefore, we further investigated the anticancer effect of niclosamide using a-well-established subcutaneous 4T1 tumor model in female BALB/c mice. The results showed that niclosamide inhibited the tumor growth by 43% at the dose of 20 mg/kg. However, it was found that niclosamide at the dose of 10 mg/kg blocked lung metastasis without statistical significance. Based on the results, we doubt that the modest inhibitory rate and lung metastasis *in vivo* might be due to the low dose of niclosamide and its pool bioavailability. Therefore, in depth studies should be still needed to enhance its antitumor and antimetastatic activities *in vivo*. Fortunately, the *in vivo* results also exhibited that niclosamide can reduce expression of Ki67 and increase expression of cleaved caspase-3 in tumor cells compared with vehicle treated group. Furthermore, accumulating evidence suggests that Stat3 plays an important role in up-regulating VEGF gene expression and inducing tumor angiogenesis under both physiological and pathological conditions [16], [42]. Inhibition of Stat3 can result in the suppression of tumor angiogenesis, which was also observed in tumor tissues treated with niclosamide in this study. These results suggested that niclosamide may have a role in the treatment of angiogensis.

Breast cancer is progressing toward increasingly malignant behavior in tumorigenic and metastatic stages. In the process of metastasis, tumor cells will leave the primary tumor in breast and metastasize to distant sites (lung, liver and lymph node) where they establish secondary tumors [36], [43]. Moreover, it has been reported that Src/FAK/MMP (Matrix metalloproteinase) involved pathway is critical for breast cancer cell migration and invasion [34], [44]. Therefore inhibition of the step is a promising approach to antitumor and antimetastasis treatment [34]. In this study, our observations indicated that niclosamide can inhibit breast cancer migration and invasion *in vitro* by down-regulating FAK phosphorylation at tyrsion residue 925 and Src- phosphorylation at tyrsion residue 416. Furthermore, in our animal experiments, administrations of niclosamide at the dose of 20 mg/kg significantly inhibited breast tumor metastasis to lung (Figure 5D). Overall, these results suggested that niclosamide may be a potential candidate for treating breast cancer metastasis.

A recent studies showed that Stat3 is frequently activated not only in diverse cancer cells by common oncogenic pathways, but also in tumor endothelial and myeloid cells, including Gr1^+^/CD11b^+^ (MDSCs) and tumor-associated macrophages, mediating immune suppression [34], [45]. Meanwhile, myeloid cells and other immune cells are critical components of the tumor microenvironment, and an excess of MDSCs can promote tumor angiogenesis and influence antitumor immune responses. Therefore, MDSCs play a central role in carcinoma progression in tumor-bearing mice and cancer patients [46]. In this study, our data showed that the treatment of mice with niclosamide caused a significant decrease in the number of MDSCs in tumors compared with that of vehicle treated group. It is therefore conceivable that blocking Stat3 signaling with niclosamide *in vivo* can induce immune-mediated antitumor effects.

In conclusion, the results presented here are to our knowledge the first study to demonstrate that niclosamide can inhibit breast cancer cell growth by inducing apoptosis, and block cell migration and invasion. In addition, niclosamide suppressed the breast tumor growth without significant toxicity. Moreover, niclosamide could enhance antitumor immunity and inhibit lung metastasis by reducing the number of Gr1^+^/CD11b^+^ (MDSCs) in tumors. Therefore, our studies provided strong evidence that niclosamide as a candidate of antibreast cancer drug is worth being further investigated.
